# Dialogue between nutrition and reproduction: preliminary exploration of sperm quality response to high-fat diet in mice

**DOI:** 10.3389/fcell.2025.1640714

**Published:** 2026-01-12

**Authors:** Yali Jiang, Wenge Zhang

**Affiliations:** 1 Ili & Jiangsu Joint Institute of Health, The Friendship Hospital of Ili Kazakh Autonomous Prefecture, Ili, Xinjiang, China; 2 The Friendship Hospital of Ili Kazakh Autonomous Prefecture, Ili, Xinjiang, China

**Keywords:** apoptosis, high-fat diet, mice, oxidative stress, semen quality

## Abstract

**Background:**

High-fat diets are known to affect semen quality, potentially through metabolic and cellular stress mechanisms.

**Objectives:**

This study investigates the impact of a high-fat diet on semen quality in male mice and identifies the underlying mechanisms involved.

**Materials and methods:**

Thirty male C57BL/6J mice were randomly assigned to a control diet (CD) or HFD for 12 weeks. Sperm parameters and fertility were assessed by computer-assisted sperm analysis and mating trials. Testicular oxidative stress indices, autophagy- and apoptosis-related proteins were analyzed by biochemical assays and Western blot. Mitophagy (LC3B–Grp75 co-localization), tissue morphology (H&E, TUNEL, electron microscopy) and sperm DNA damage (DNA fragmentation index, 8-hydroxy-2′-deoxyguanosine [8-OHdG]) were further evaluated. Serum lipids and testosterone were measured by enzymatic assays and ELISA.

**Results:**

HFD mice developed obesity, dyslipidemia, reduced testosterone, marked decreases in sperm count, progressive motility and normal morphology, and complete loss of fertility. Region-specific molecular alterations were observed along the reproductive tract: in epididymal caput, Beclin-1 and LC3B were upregulated and p62 downregulated, indicating enhanced autophagic flux; in cauda epididymis, pro-apoptotic markers increased and Bcl-2 decreased, consistent with augmented apoptosis; in testis, ATG5, Beclin-1, caspase-3 and Apaf-1 were elevated. HFD also increased testicular ROS and MDA, reduced SOD activity and mitochondrial membrane potential, and elevated sperm apoptosis, DNA fragmentation and 8-OHdG.

**Conclusion:**

HFD induces a pathogenic cascade—oxidative stress, disrupted autophagy/mitophagy, and mitochondrial apoptosis-that collectively impair spermatogenesis and male fertility. Therapeutic strategies targeting antioxidant defenses, autophagic flux, and mitophagy may ameliorate obesity-related reproductive dysfunction.

## 1 Introduction

Male infertility has been recognized as a significant global public health issue, with impaired spermatogenesis being one of its primary causes. This dysfunction is often characterized by decreased sperm concentration and motility, increased DNA fragmentation, and morphological abnormalities, all of which are critical determinants of male reproductive capacity. In addition to genetic and hormonal regulation, growing evidence indicates that environmental and lifestyle factors profoundly influence spermatogenesis and compromise male reproductive health. Recent studies have shown that long-term consumption of a high-fat diet (HFD) leads to obesity, metabolic syndrome, and decreased testosterone levels, accompanied by increased apoptosis of spermatogenic cells, elevated oxidative stress, mitochondrial dysfunction, and altered autophagy pathways (Lalrinzuali et al., 2023; Falvo et al., 2023; Chen et al., 2025; Marei et al., 2025). Specifically, this is manifested by activation of Caspase-3, release of cytochrome c, accumulation of reactive oxygen species (ROS), and abnormal expression of LC3-II, Beclin-1, and p62, suggesting a coordinated dysregulation of apoptosis, oxidative stress, and autophagy. Beyond diet, other environmental factors such as endocrine-disrupting chemicals, air pollution, heavy metals, heat stress, and microplastics have also been implicated in the disruption of spermatogenesis (Xia et al., 2022; Zhou et al., 2023; Kaltsas et al., 2025; Gautam et al., 2024; Daniels and Berger Eberhardt, 2024). Recent IJMS reports further indicate that dibutyl phthalate (DBP) perturbs ER/AR signaling, while glyphosate activates ERα/ERβ and induces mitochondrial dysfunction, strengthening the mechanistic link between endocrine disruptors and impaired spermatogenesis (Mileo et al., 2023; Chianese et al., 2024). For example, bisphenol A induces endoplasmic reticulum (ER) stress and ROS-mediated apoptosis via the GRP78–PERK signaling pathway; long-term exposure to PM2. 5 impairs sperm motility and induces DNA damage; and heavy metals like cadmium and lead reduce antioxidant enzyme activity and interfere with Leydig and Sertoli cell functions. Moreover, metabolic disorders arising from unhealthy lifestyles—such as insulin resistance and chronic inflammation—have also been validated as key factors affecting reproductive health. Insulin resistance impairs the hypothalamic–pituitary–gonadal (HPG) axis by decreasing FSH and LH secretion, thereby inhibiting testosterone production and spermatogenesis (Balcázar-Hernández et al., 2023; Hsia et al., 2022). Environmental pollutants and obesity may further influence spermatogenesis via epigenetic modifications, including DNA methylation, histone modification, and altered small noncoding RNA profiles in sperm, potentially exerting transgenerational effects (Nikolaou et al., 2024; Ameratunga et al., 2023). Although many studies have independently elucidated the detrimental effects of these factors on male fertility, few have systematically explored how HFD, environmental exposure, and metabolic disorders synergistically modulate apoptosis, oxidative stress, and autophagy signaling. Most existing literature has focused on isolated sperm parameters or single molecular pathways, while integrated analyses involving mTOR/AMPK signaling, mitophagy markers, autophagic flux, and key apoptotic proteins remain limited. To address these gaps, this study established a high-fat diet-induced mouse model to comprehensively evaluate sperm quality and testicular histopathology. By assessing oxidative stress, autophagy, and apoptosis-related markers, we aim to explore their crosstalk and underlying mechanisms. Through a multi-dimensional analysis of sperm motility, mitochondrial membrane potential, ROS levels, DNA fragmentation index, and protein expression profiling, this study seeks to uncover potential mechanistic links in the nutrition–reproduction interface.

## 2 Materials and methods

### 2.1 Materials

#### 2.1.1 Diets

The experimental design was structured to compare the effects of a standard diet versus a high-fat diet on male C57BL/6J mice. The Standard Diet Group was provided with a diet comprising a macronutrient energy ratio of 10% fat, 20% protein, and 70% carbohydrates, reflecting a balanced nutritional profile typical of standard laboratory diets. This composition ensures a low-fat intake, aligning with the dietary requirements for maintaining normal physiological functions in mice. Conversely, the High-Fat Diet Group received a diet formulated to contain a significantly higher fat content of 60%, with the remaining energy derived from 20% protein and a minimal 10% carbohydrates. This dietary composition is designed to mimic excessive fat consumption, commonly associated with obesity in humans, and to investigate its impact on various physiological and biochemical parameters in mice. After a 12-week dietary regimen, the mice subjected to the high-fat diet exhibited a marked increase in body weight compared to their counterparts on the standard diet. The weight gain observed in the High-Fat Diet Group was both significant and substantial, indicating successful induction of an obesity model in these mice.

#### 2.1.2 Chemicals chosen as obeseals

The high-fat diet was obtained from Jiangsu Synergy Pharmaceutical and Bioengineering Co., Ltd., China. Glutaraldehyde and formaldehyde for tissue fixation were sourced from Chengdu McLean Biotechnology Co., Ltd., and Xinjiang Mio Trading Co., Ltd., respectively. Tyrode’s buffer (batch no. PB180338) for sperm analysis was from Pronosay, China. Total RNA extraction reagent and DEPC-treated water for RNA stabilization were supplied by White Shark Biotechnology Co., Ltd., Hefei, and Shanghai Sangong Bioengineering Co., Ltd., China, respectively. RNA visualization during electrophoresis utilized reagents from Beijing QuanShiJin Biotechnology Co., Ltd., China. The RT-PCR EasyTM -SYBR Green I kit for qPCR was provided by Chengdu Fukuji Biotech Co., Ltd., China. Electrophoresis reagents, including 50× TAE and agarose, were from Shanghai Sangong Bioengineering Co., Ltd., China. Reverse transcription kits were supplied by Thermo Fisher Scientific, Ltd., Shanghai, China. Cell lysis and protein extraction used reagents from Solarbio, while Western blotting was performed with the EasySee Western blot kit from TRANS. Various biochemicals, including Tris (hydroxymethyl) aminomethane (TRIS), 1M Tris-HCL (pH 7.6), SDS powder, sodium chloride, and SDS-PAGE gel preparation kits, were used from Solarbio, Biosharp, Biofroxx, and Komio. Additional reagents, including Tween-20, protein markers, PBS buffer, and staining solutions, were sourced from Solarbio. The TUNEL Apoptosis Detection Kit, Annexin V-FITC Apoptosis Detection Kit, and other biochemical kits were from Biyuntian Biotechnology Co., Ltd., while reagents for electron microscopy were obtained from Xinjiang Kemo Trading Co., Ltd., and Hyde Venture (Beijing) Biotechnology Co., Ltd.

#### 2.1.3 Laboratory animals

A total of thirty male C57BL/6J mice, aged between 6 and 8 weeks, along with twelve female mice of the same age range, were procured from the Animal Experiment Center of Xinjiang Medical University. These animals were of clean-grade quality and had initial body weights ranging from 18 to 20 g. Upon arrival, the mice underwent a one-week acclimatization period, during which they were housed under controlled environmental conditions. The temperature was maintained at 22 °C ± 3 °C, with relative humidity levels set between 40% and 60%. The facility ensured a consistent cycle of 12 h of light followed by 12 h of darkness. Throughout their stay, the mice were provided with clean-grade bedding and had *ad libitum* access to water. The commencement of group-specific treatments was authorized under license SYXK (new) 2018-0001, following the initial week of feeding and acclimatization. All animal husbandry, care and laboratory procedures are carried out in accordance with the Guidelines for the Care and Use of Laboratory Animals and ARRIVE Checklist. By taking appropriate measures to minimize pain and stress to the animals, these experiments were approved by the local experimental Animal Ethics Committee.

### 2.2 Experimental design

Thirty male C57BL/6J mice (6–8 weeks old) were divided into two groups: standard diet (CD, n = 15) and high-fat diet (HFD, n = 15), and fed accordingly for 12 weeks. Weekly assessments included dietary intake and body mass. After the diet period, mice were co-housed with females to assess reproductive outcomes. The Standard Diet Group received a diet with 10% fat, 20% protein, and 70% carbohydrates, typical of balanced laboratory diets. The High-Fat Diet Group’s diet contained 60% fat, 20% protein, and 10% carbohydrates. Mice were fed either a control diet or a high-fat diet (HFD) for 12 weeks. The 12-week duration was selected based on previous reports showing that metabolic and reproductive alterations in HFD-fed mice typically emerge after 10–12 weeks of continuous exposure, when obesity, insulin resistance, and testicular oxidative stress become stably established. Shorter feeding periods (for instance, 4–8 weeks) can induce early metabolic changes but are often insufficient to cause significant sperm quality impairment or testicular histopathological alterations (Lang et al., 2019). Therefore, the 12-week regimen was chosen to ensure full manifestation of diet-induced obesity and its downstream reproductive effects.

### 2.3 Assessment of mouse sperm quality

Mice were anesthetized with sodium pentobarbital and euthanized by cervical dislocation. Tissues from the cauda of the epididymis were excised and placed in pre-warmed Tyrode’s buffer at 37 °C. The tissues were gently incised to release spermatozoa and incubated at 37 °C with 5% CO_2_ for 15 min. Subsequently, 5–10 µL of the sperm suspension was collected for analysis. Sperm parameters were evaluated using a computer-assisted sperm analysis (CASA) system, including sperm concentration, percentage of morphologically normal sperm, progressive and non-progressive motility, percentage of immotile sperm, total sperm count, and progressively motile sperm count.

### 2.4 RNA extraction and qRT-PCR

Total RNA was extracted separately from the epididymal caput, cauda epididymis, and testis of each mouse using TRIzol reagent (Invitrogen, USA) according to the manufacturer’s instructions. The epididymis was dissected under a stereomicroscope, and the caput and cauda regions were isolated based on their anatomical features. The caput mainly represents the site of sperm maturation, whereas the cauda serves as the sperm storage region. Including both regions allowed us to distinguish regional gene expression differences along the epididymis under high-fat diet (HFD) conditions. In addition, testicular tissue was analyzed to evaluate upstream effects on spermatogenesis and germ cell apoptosis. RNA purity and concentration were assessed using a NanoDrop 2000 spectrophotometer (Thermo Fisher Scientific), and cDNA synthesis was performed using a reverse transcription kit (Takara, Japan). Quantitative PCR was performed with SYBR Green Master Mix (Bio-Rad, USA), and relative expression levels were calculated using the 2^−ΔΔCt^ method. The reaction volume was 20 μL, consisting of 10 µL SYBR Green Master Mix, 1 µL each of forward and reverse primers, 2 µL of cDNA template, and 6 µL of nuclease-free water. The cycling program was as follows: 95 °C for 30 s for initial denaturation, followed by 40 cycles of 95 °C for 5 s and 60 °C for 30 s, with a final melting curve analysis to verify product specificity.

### 2.5 Western blot detection of mitochondrial and autophagy marker protein expression in mouse sperm

Sperm samples from CD and HFD groups were centrifuged at 2,000 × g for 5 min, washed with PBS, and lysed in ice-cold RIPA buffer supplemented with 1 mM PMSF (Solarbio, China). Total protein was quantified using a BCA Protein Assay Kit (Thermo Fisher, Cat# 23225). Equal amounts (20 μg) of protein were boiled in 4× SDS sample buffer at 100 °C for 10 min, separated by 12% SDS-PAGE, and transferred to PVDF membranes (Millipore, IPVH00010) at 350 mA for 90 min. Membranes were blocked with 5% BSA in TBST (0.1% Tween-20) for 1 h at room temperature and then incubated overnight at 4 °C with the following primary antibodies:Bax (1:1,000, Cell Signaling Technology, CST#2772), Bcl-2 (1:1,000, CST#3498), Caspase-3 (1:1,000, CST#9662), Caspase-8 (1:1,000, CST#9746), Caspase-9 (1:1,000, CST#9508), Mn SOD (1:1,000, Abcam, ab13533), Beclin-1 (1:1,000, CST#3495), LC3B (1:1,000, CST#3868), β-Actin (1:2,000, CST#3700) as the internal control. After washing (3 × 5 min in TBST), membranes were incubated with HRP-conjugated goat anti-rabbit IgG (1:5,000, CST#7074) for 1 h at room temperature. Detection was performed using an ECL substrate (Thermo Fisher, Cat# 32106), and signals were imaged on a Bio-Rad ChemiDoc MP system. Band intensities were analyzed with ImageJ software, and target protein levels were normalized to β-Actin.

### 2.6 HE staining observation of mouse testicular tissues

Testicular and cauda epididymis epididymis tissues were fixed in formaldehyde, embedded in paraffin, and sectioned. Sections were dewaxed, hydrated, stained with hematoxylin, differentiated, and blued. Eosin staining followed, and sections were dehydrated, cleared in xylene, air-dried, and mounted for microscopic observation.

### 2.7 Immunofluorescence assay for co-localized expression of LC3B, p62, LC3B+Grp75 in mouse semen

Sperm smears were air-dried and fixed in 4% paraformaldehyde (Servicebio, G1101) for 30 min at room temperature. Samples were permeabilized with 0.3% Triton X-100 (Sigma, T8787) for 5 min, blocked with 5% BSA for 1 h, and incubated overnight at 4 °C with the following primary antibodies:LC3B (1:200, CST#3868), p62/SQSTM1 (1:200, Abcam, ab56416), Grp75 (1:200, Abcam, ab2799). After washing in PBS, sections were incubated with FITC-conjugated secondary antibody (1:500, Beyotime, A0562) for 1 h at room temperature in the dark. Nuclei were counterstained with DAPI (1 μg/mL, Sigma, D9542). After mounting with anti-fade medium (Beyotime, P0126), images were captured using a Leica TCS SP8 confocal microscope. Mean fluorescence intensity of target proteins was quantified using ImageJ software. For each mouse, two sperm smears were prepared from the cauda epididymis to ensure consistency and reproducibility. A total of 12 mice per group were analyzed, resulting in 24 smears per group. At least 200 spermatozoa per smear were evaluated under a light microscope for morphology, motility, and fragmentation indices, For both CD and HFD groups, sperm released from the cauda epididymis were adjusted to a standardized working concentration of 2.5 × 10^6^ sperm/mL (range 2.0∼3.0 × 10^6^sperm/mL). and the mean of the two smears was used for statistical analysis.

### 2.8 Colorimetric TUNEL apoptosis detection in mouse semen

Sperm samples were washed, fixed, and permeabilized. Endogenous peroxidase was inactivated, and biotin labeling was performed. Samples were incubated with streptavidin-HRP, followed by DAB color development. Apoptosis was assessed microscopically.

### 2.9 Flow cytometry to detect apoptosis rate in mouse semen cells

Sperm samples were washed and stained with Annexin V/FITC and PI. JC-1 staining and ROS detection with DCFH-DA were performed. Acridine Orange staining followed, and samples were analyzed using flow cytometry.

### 2.10 Detection of blood lipid and testosterone levels in mice

Serum samples from CD and HFD groups were analyzed for triglycerides, total cholesterol, LDL, HDL, and testosterone using biochemical assays and ELISA to assess metabolic and hormonal changes affecting male fertility.

### 2.11 Transmission electron microscopy observation of testicular tissue

Testicular tissues were fixed in glutaraldehyde, dehydrated in ethanol, and embedded in epoxy resin. Ultrathin sections were double-stained with uranyl acetate and lead citrate. Sections were examined under a transmission electron microscope to observe ultrastructural changes.

### 2.12 Activity of reactive oxygen species, lipid peroxidation products, and antioxidant enzymes in mouse testicular cells

Single-cell suspensions were prepared by enzymatic digestion of testicular tissue (1:10 w/v in trypsin; 37 °C, 30 min), filtration through a 400-mesh nylon sieve, and two PBS washes (2,000 × g, 4 °C, 3 min). For ROS detection, cells were incubated with 10 µM DCFH-DA in PBS (1:1,000 v/v) at 37 °C for 30 min in the dark, washed twice, and fluorescence (Ex488/Em525 nm) was quantified in triplicate on a multimode plate reader. Malondialdehyde (MDA) content was determined by the thiobarbituric acid reactive substances (TBARS) method: tissue homogenates (1:10 w/v) were reacted with TBA reagent at 100 °C for 30 min, centrifuged (10,000 × g, RT, 10 min), and absorbance was read at 450, 532 and 600 nm. SOD activity was assayed using a WST-1 based kit where supernatants (1:10 w/v homogenates) were incubated with sequential reaction reagents I–V at 37 °C for 30 min, and the inhibition of WST-1 reduction was measured at 560 nm. All assays were performed in triplicate, and values were normalized to protein concentration (BCA assay).

### 2.13 Detection of autophagy-related proteins

Testicular tissues (n = 3 per group) were homogenized in ice-cold RIPA buffer containing 1 mM PMSF, lysed on ice for 30 min, and centrifuged (12,000×g, 4 °C, 15 min) to obtain total protein. Protein concentrations were determined by BCA assay (Thermo Fisher, Cat. 23225), and 20 µg of each lysate was mixed with 4 Laemmli buffer, denatured at 100 °C for 10 min, and separated on 12% SDS–PAGE gels (80 V for stacking, 120 V for resolving). Proteins were then transferred to 0.45 μm PVDF membranes (Millipore, IPVH00010) at 350 mA for 90 min, blocked in 5% BSA-TBST (0.1% Tween-20) for 1 h at room temperature, and incubated overnight at 4 °C with primary antibodies against ATG5 (1:1,000, CST #12994), Beclin-1 (1:1,000, CST #3495), LC3B (LC3Ⅱ/LC3Ⅰ, 1:1,000, CST #3868), and β-Actin (1:2,000, CST #3700). After washing (3 × 5 min in TBST), membranes were incubated with HRP-conjugated secondary antibodies (1:5,000) for 1 h at room temperature, washed again (3 × 5 min), and developed with ECL substrate (Thermo Fisher, Cat. 32106) for 2 min. Blots were imaged on a ChemiDoc MP system (Bio-Rad), and band intensities were quantified using ImageJ, with target proteins normalized to β-Actin.

### 2.14 Detection of key proteins in the apoptotic signaling pathway in testicular tissue

Cytosolic and mitochondrial fractions were isolated from testicular homogenates using a cell fractionation kit (Thermo Fisher, Cat. 89874). Protein concentrations of each fraction were determined by the BCA method, and 20 µg of cytosolic protein was subjected to 12% SDS–PAGE followed by transfer to PVDF membranes at 350 mA for 90 min. Membranes were blocked in 5% BSA–TBST for 1 h at room temperature, then incubated overnight at 4 °C with primary antibodies (1:1,000). After three 5-min washes in TBST, membranes were incubated with HRP-conjugated secondary antibodies (1:5,000) for 1 h at room temperature and developed with ECL for 2 min. Band intensities of cytosolic Cytochrome c, Cleaved Caspase-3, and Apaf-1 were quantified by ImageJ and normalized to β-Actin.

### 2.15 Detection of key proteins in the apoptotic signaling pathway in sperm samples

Cytosolic and mitochondrial fractions were isolated from testicular homogenates using a cell fractionation kit (Thermo Fisher, Cat. 89874). Protein concentrations of each fraction were determined by the BCA method, and 20 µg of cytosolic protein was subjected to 12% SDS–PAGE followed by transfer to PVDF membranes at 350 mA for 90 min. Membranes were blocked in 5% BSA-TBST for 1 h at room temperature, then incubated overnight at 4 °C with primary antibodies (1:1,000). After three 5-min washes in TBST, membranes were incubated with HRP-conjugated secondary antibodies (1:5,000) for 1 h at room temperature and developed with ECL for 2 min. Band intensities of cytosolic Cytochrome c, Cleaved Caspase-3, and Apaf-1 were quantified by ImageJ and normalized to β-Actin.

### 2.16 Measurement of 8-OHdG in spermatozoa

After sperm isolation and washing, DNA was extracted using a commercial DNA isolation kit. The concentration of oxidative DNA damage was assessed by measuring 8-hydroxy-2′-deoxyguanosine (8-OHdG) levels using a competitive ELISA kit following the manufacturer’s instructions. Absorbance was read at 450 nm using a microplate reader. Each sample was run in triplicate, and results were normalized to total DNA content. Data are expressed as ng 8-OHdG per mg DNA.

### 2.17 Statistical methods

All data were entered in Excel and analyzed and plotted using GraphPad Prism7. 00 software package. Comparisons between groups were made using the t-test. *P* < 0.05was considered statistically significant and marked as *; *: *P* < 0.05, **: *P* < 0.01, ***: *P* < 0.001.

## 3 RESULTS

### 3.1 Dietary intake and body mass changes in mice across groups

During weeks 1–3, no significant difference in body weight was observed between mice fed a Control Diet (CD) and those on a High-Fat Diet (HFD) (*P* > 0.05). However, from weeks 3–6, mice on the HFD showed a significantly greater weight increase compared to the CD group (*P* < 0.001). Despite consuming less food, the HFD group gained more weight, highlighting the impact of diet composition on weight gain (*P* < 0.001). Notably, despite lower overall food intake, the HFD group gained more weight, underscoring the obesogenic impact of dietary fat content. These findings confirm the successful establishment of a diet-induced obesity model in mice.

### 3.2 Litter production comparison between groups

In the reproductive aspect of the study, a stark contrast was observed in the fertility outcomes between the two groups. Female mice co-housed with male mice from the High-Fat Diet (HFD) group did not conceive, indicating a significant adverse effect of the HFD on male fertility. Conversely, female mice paired with males from the Control Diet (CD) group successfully produced a total of 17 pups. This clear disparity highlights the potential negative impact of high-fat dietary intake on male reproductive capability.

### 3.3 Expression of apoptosis- and autophagy-related genes in the epididymal caput, cauda, and testis

**FIGURE 1:**
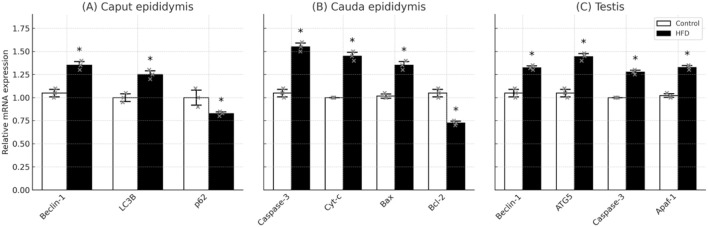
Differential mRNA expression of autophagy- and apoptosis-related genes in the epididymal caput, cauda, and testis of mice fed a control diet (CD) (n = 3) or high-fat diet (HFD) (n = 3). **(A)** Relative mRNA expression levels of Beclin-1, LC3B, and p62 in the epididymal caput. HFD mice showed mild upregulation of autophagy markers (Beclin-1, LC3B) and downregulation of p62, suggesting activation of autophagic flux in the early epididymal region. **(B)** Relative mRNA expression levels of Caspase-3, Cyt-c, Bax, and Bcl-2 in the cauda epididymis. Marked elevation of pro-apoptotic genes (Caspase-3, Cyt-c, Bax) and reduced Bcl-2 expression indicated enhanced apoptosis associated with sperm storage and motility impairment. **(C)** Relative mRNA expression levels of Beclin-1, ATG5, Caspase-3, and Apaf-1 in the testis. HFD mice exhibited significant activation of both autophagy and apoptosis pathways, consistent with histological evidence of germ cell loss. Data are expressed as mean ± standard deviation (x̄ ± s). Statistical comparisons between CD and HFD groups were performed using independent-samples t-tests. P < 0.05 was considered statistically significant (*P* < 0.05, *P < 0.01 vs. CD group).

**FIGURE 2:**
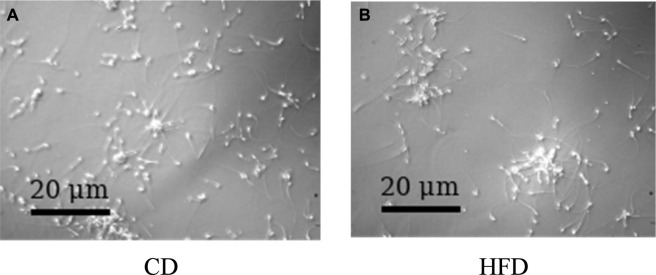
CASA-based sperm motility assessment in mice fed a control diet (CD) or high-fat diet (HFD). **(A)** Representative sperm motion trajectory from a control diet (CD) mouse showing long, continuous, and directional paths, indicating vigorous forward motility and normal sperm activity. **(B)** Representative sperm motion trajectory from a high-fat diet (HFD) mouse showing short, fragmented, and irregular paths, suggesting reduced motility and impaired trajectory linearity. Data were analyzed using a computer-assisted sperm analysis (CASA) system. The differences in motility parameters between groups were further quantified in subsequent figures.

Compared with the control diet (CD) group, high-fat diet (HFD) mice exhibited significant transcriptional alterations in both the epididymis and testis (Figure 2). In the epididymal caput, the mRNA expression of Beclin-1 and LC3B was moderately increased, while p62 expression was downregulated, suggesting early activation of autophagic flux. In contrast, the cauda epididymis displayed a more pronounced upregulation of Caspase-3, Cyt-c, and Bax, accompanied by a decrease in Bcl-2, indicating enhanced apoptotic activity during sperm storage. In the testis, the expression of autophagy-related genes (Beclin-1, ATG5) and apoptosis regulators (Caspase-3, Apaf-1) was markedly elevated in the HFD group compared with the CD group (*P* < 0.01). These changes coincided with histological observations of germ cell loss and seminiferous tubule degeneration, implying that long-term high-fat feeding induced both oxidative stress and autophagy-mediated cell death in testicular tissue. Collectively, these findings indicate region-specific molecular responses within the male reproductive tract, where the caput epididymis undergoes compensatory autophagic adaptation, the cauda shows apoptotic activation associated with sperm impairment, and the testis demonstrates combined autophagy-apoptosis dysregulation under HFD exposure Figure 1.

### 3.4 Sperm quality comparison between groups

The study found significant differences in sperm quality between dietary groups: Total sperm count and motility were lower in the High-Fat Diet (HFD) group compared to the Control Diet (CD) group, indicating a negative impact on sperm quantity and motility. Progressive motility (PR%) and sperm concentration (SC) were also reduced in the HFD group, highlighting the adverse effects on sperm health. The HFD group had a lower percentage of sperm with normal morphology, affecting structural integrity, and a higher percentage of non-progressive motility (NP%), indicating poorer motility quality. Although immobility (IM%) was higher in the HFD group, the difference was not statistically significant (*P* > 0.05). These findings are illustrated in Figure 2 and detailed in Table 1.

**TABLE 1:** Comparison of results of sperm routine parameters among groups of mice ( $\bar{x}$ ± s).

Groups	CD group	HFD group	*t*	*P*
Total number of spermatozoa (total)	691.5 ± 142.5	417.5 ± 44.57**	4.494	0.0012
Total number of forward-moving spermatozoa (progressive)	111.3 ± 19.65	56.67 ± 6.919***	6.427	<0.0001
Percentage of forward-moving sperm (PR%)	16.18 ± 0.6,078	13.61 ± 1.065***	5.128	0.0004
Semen concentration (10^6^/mL)	83.89 ± 8.244	55.67 ± 6.645***	6.529	<0.0001
Percentage of normal morphology spermatozoa (%)	65.02 ± 3.662	54.2 ± 6.979**	3.362	0.0072
Percentage of non-forward-moving sperm (NP%)	4.733 ± 0.5187	6.255 ± 1.301*	2.662	0.0238
Percentage of immobile sperm (IM%)	79.09 ± 1.018	80.1 ± 2.346	0.3524	0.9753

Data are expressed as mean ± standard deviation ( $\bar{x}$ ± s). The t value represents the statistic from an independent-samples t-test, and *P* indicates the corresponding probability value. Significance levels are indicated as *P* < 0.05, *P < 0.01, and **P < 0.001. *** indicates a statistically significant difference at P < 0.001 compared with the control diet (CD) group.

### 3.5 Comparison of gene expression related to apoptosis and oxidative stress

**FIGURE 3:**
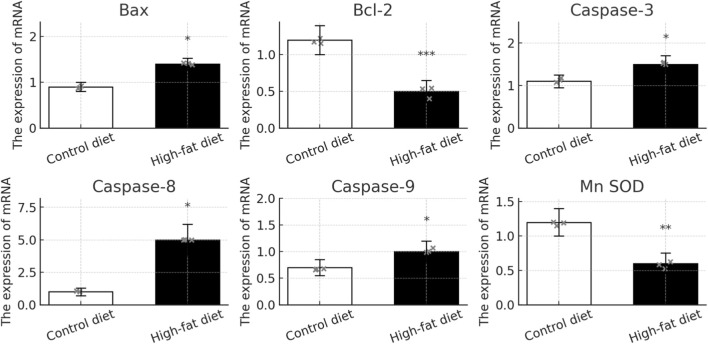
qRT-PCR analysis of BAX and Caspase-3 mRNA in epididymal tissue. Total RNA was extracted from epididymal head and cauda epididymis of CD (n = 3) and HFD (n = 3) mice using TRIzol. After reverse transcription (PrimeScript RT Master Mix, Takara), qPCR was performed on a Bio-Rad CFX96 with SYBR Green I. GAPDH was internal control; primer sequences in Table 1. Cycling: 94 °C 3 min; 45× (94 °C 5 s, 60 °C 20 s). Note: *: *P* < 0.05, **: *P* < 0.01, ***: *P* < 0.001 vs. CD diet.

The study examined gene expression levels related to apoptosis and oxidative stress in sperm from Control Diet (CD) and High-Fat Diet (HFD) groups, revealing significant differences. Pro-apoptotic genes (Bax, Caspase-3, -8, -9) were significantly higher in the HFD group, indicating increased activation of apoptotic pathways, potentially reducing sperm quality. Conversely, anti-apoptotic and antioxidant genes (Bcl-2, Mn SOD) were significantly lower in the HFD group, suggesting a reduced ability to resist cell death and oxidative damage. These findings (*P* < 0.05) highlight the diet’s impact on molecular pathways regulating sperm health, with detailed results in Table 2 and Figure 3.

**TABLE 2:** Gene expression in each group of cells ( $\bar{x}$ ± s).

Clusters	CD group	HFD group	*t*	*P*
Bax	1.057 ± 0.060	1.481 ± 0.132*	2.924	0.0431
Bcl-2	1.011 ± 0.067	0.554 ± 0.018*	6.593	0.0027
Caspase-3	1.007 ± 0.052	1.203 ± 0.038*	3.058	0.0377
Caspase-8	1.106 ± 0.0001	6.699 ± 0.002*	3.385	0.0277
Caspase-9	1.005 ± 0.052	1.184 ± 0.038*	2.784	0.0496
Mn SOD	1.004 ± 0.051	0.748 ± 0.014	4.833	0.0084

Data are expressed as mean ± standard deviation ( $\bar{x}$ ± s). Statistical comparisons between the two groups were performed using the independent-samples t-test. The t value represents the test statistic, and *P* denotes the probability value. A *P* < 0.05 was considered statistically significant. Significance levels are indicated as *P* < 0.05, *P < 0.01, and **P < 0.001.

### 3.6 Comparison of apoptosis, autophagy, and oxidative stress marker expression

**FIGURE 4:**
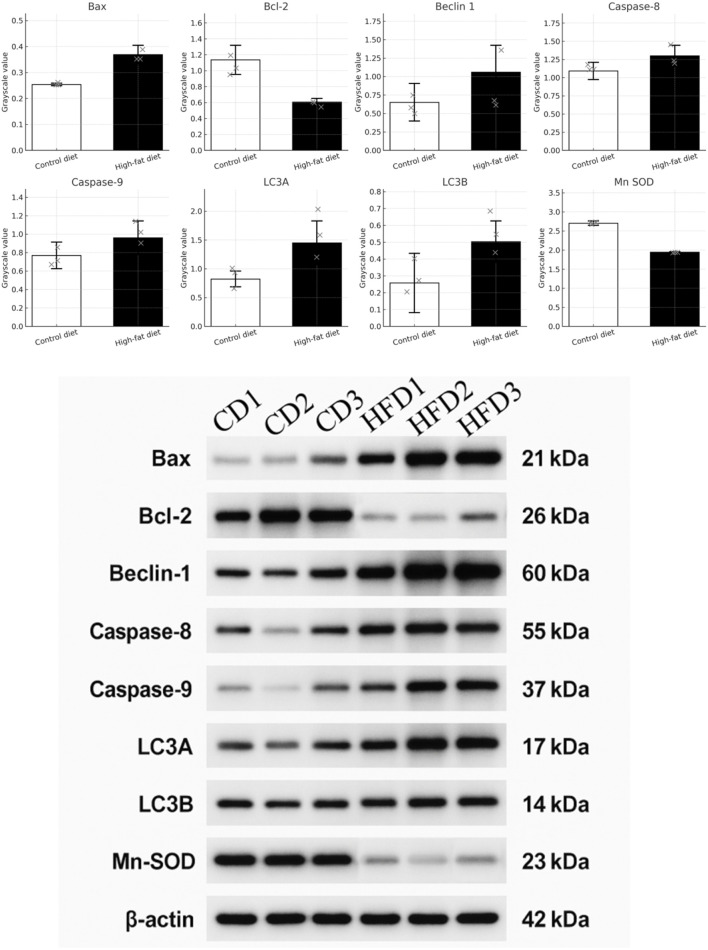
Western blot analysis and quantification of apoptosis, autophagy, and oxidative stress markers. Proteins were extracted from sperm samples of the control diet (CD, n = 3) and high-fat diet (HFD, n = 3) groups using RIPA buffer. Equal amounts of protein (20 μg) were separated via 12% SDS–PAGE and transferred to PVDF membranes (350 mA, 90 min). Membranes were blocked with 5% BSA and incubated overnight at 4 °C with primary antibodies against Bax, Bcl-2, Beclin-1, Caspase-8, Caspase-9, LC3A/B, Mn SOD (all 1:1,000, CST), and β-Actin (1:2,000). After incubation with HRP-conjugated secondary antibodies (1:5,000) and ECL detection, protein bands were visualized and quantified using ImageJ. Densitometric values were normalized to β-Actin. Quantitative results are shown as bar graphs for each marker, illustrating the differential expression between groups. Note: *: *P* < 0.05, **: *P* < 0.01, ***: *P* < 0.001 vs. CD.

The study compared gene expression levels related to apoptosis, autophagy, and oxidative stress between mice fed a Control Diet (CD) and those on a High-Fat Diet (HFD), revealing significant differences:Increased Expression in HFD Group:Apoptosis: Bax, Caspase-8, Caspase-9Autophagy: Beclin 1, LC3A, LC3B These findings suggest enhanced apoptotic and autophagic activity in the HFD group, contributing to impaired sperm quality. Decreased Expression in HFD Group:Apoptosis Inhibition: Bcl-2 Oxidative Stress Defense: Mn SOD Lower levels of these genes indicate a reduced capacity to resist oxidative damage and apoptosis. Non-detected Proteins:ATG5: Essential for autophagosome formationCaspase-3: Key effector in apoptosis The absence suggests these pathways may not be significantly impacted by diet or were below detectable levels. The statistically significant differences (*P* < 0.05) highlight the molecular impacts of a high-fat diet on male reproductive health. Results are shown in Figure 4 and detailed in Table 3.

**TABLE 3:** Gray scale values of Bax, Bcl-2, Beclin 1, Caspase-8, Caspase-9\LC3B/LC3A, LC3B/β-Actin, Mn SOD protein bands in each group ( $\bar{x}$ ±s, n = 3).

Clusters	CD group	HFD group	*t*	*P*
Bax	0.254 ± 0.005	0.369 ± 0.036*	3.14	0.0349
Bcl-2	1.136 ± 0.183	0.607 ± 0.044*	2.813	0.0482
Beclin 1	0.652 ± 0.255	1.060 ± 0.364	0.9197	0.4098
Caspase-8	1.092 ± 0.118	1.301 ± 0.144	1.127	0.323
Caspase-9	0.769 ± 0.144	0.960 ± 0.182	0.8245	0.456
LC3A	0.823 ± 0.137	3.381 ± 0.449**	5.446	0.0055
LC3B	0.258 ± 0.176	1.205 ± 0.503***	22.67	<0.0001
Mn SOD	2.705 ± 0.057	1.946 ± 0.011**	13.15	0.0002

Data are expressed as mean ± standard deviation ( $\bar{x}$ ± s). The t value represents the statistic from an independent-samples t-test, and *P* denotes the corresponding probability value. A *P* < 0.05 was considered statistically significant. Significance levels are indicated as *P* < 0.05, *P < 0.01, and **P < 0.001.

### 3.7 Histological and ultrastructural alterations in testicular tissue

**FIGURE 5:**
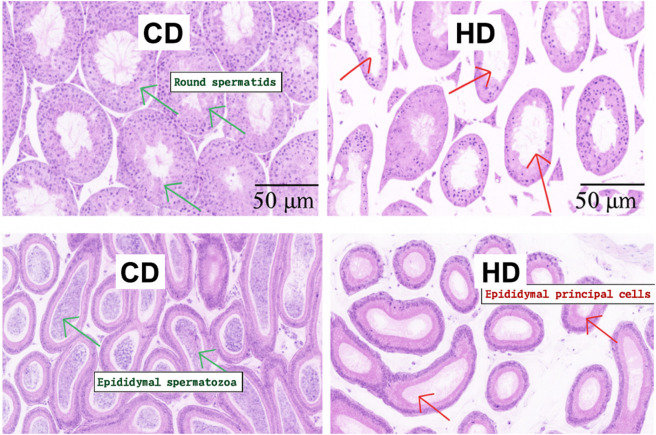
H&E staining of testicular tissue/cauda epididymis (×400/×600; scale bar 50 μm). Testes from CD (n = 3) and HFD (n = 3) mice were fixed in 10% formalin, paraffin-embedded, sectioned (5 µm), dewaxed, rehydrated, and stained with hematoxylin & eosin.

**FIGURE 6:**
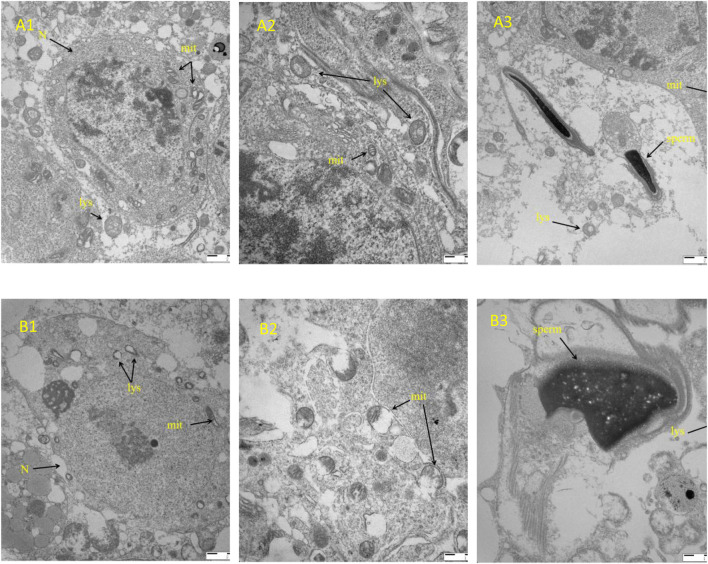
Transmission electron microscopy of testicular ultrastructure. Testicular samples from CD and HFD mice (n = 3 each) were fixed in 2.5% glutaraldehyde, post-fixed in osmium tetroxide, dehydrated in graded ethanol, embedded in epoxy resin, and sectioned (70 nm). Sections were stained with uranyl acetate and lead citrate and observed under TEM at 10,000× and 20,000×. Arrows indicate mitochondrial vacuolization (mit) and chromatin condensation (N). Note: A: CD group; B: HFD group; N: nucleus; mit: mitochondria; lys: lysosomes mitochondria; sperm: spermatozoa.

**FIGURE 7:**
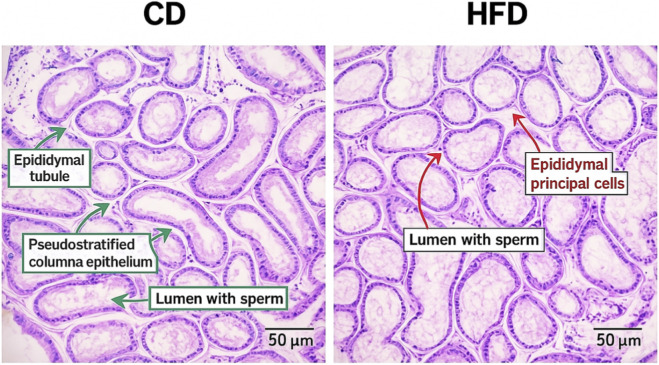
H&E staining of the epididymal caput in control diet (CD) and high-fat diet (HFD) mice (×400; scale bar = 50 µm). Representative hematoxylin and eosin–stained sections of the epididymal caput showing tightly arranged epididymal tubules lined with pseudostratified columnar epithelium and lumens containing spermatozoa. CD mice display normal epithelial height and stereocilia morphology, whereas HFD mice show mildly reduced epithelial height and subtle alterations in epididymal principal cells, with no marked differences in overall tubule architecture between groups (n = 3 per group).

Hematoxylin and eosin (H&E) staining and transmission electron microscopy (TEM) revealed consistent structural disruptions in testicular tissue of mice fed a high-fat diet (HFD) compared to the control diet (CD) group. In the H&E-stained sections, the HFD group exhibited a marked reduction in spermatogenic cell layers, disorganization of seminiferous epithelium, enlarged tubular lumina, and reduced numbers of mature spermatozoa within the epididymal lumen. In contrast, the CD group showed intact tubular architecture and abundant luminal spermatozoa. These histological changes suggest impaired spermatogenesis under HFD conditions (Figure 5). TEM analysis provided complementary ultrastructural insights. Testicular tissues from the HFD group displayed swollen and vacuolated mitochondria, decreased electron density of nuclear chromatin, and numerous interstitial vacuoles in Leydig and Sertoli cells. The number of mitochondria appeared reduced, and mitochondrial cristae were often fragmented or lost. In contrast, the CD group exhibited normal mitochondrial morphology, intact nuclear chromatin, and well-organized cellular ultrastructure (Figure 6). The combined histological and ultrastructural findings strongly indicate that a high-fat diet leads to both cellular and subcellular damage in the testicular microenvironment, which may underlie the observed impairments in sperm production and fertility. Representative H&E images of seminiferous tubule. Round spermatids (arrows) are identified in the adluminal compartment by small spherical nuclei with finely dispersed, lightly condensed chromatin and early acrosomal caps; elongated spermatids (arrowheads) show condensed, elongated nuclei with a well-defined acrosomal cap facing the lumen. At least five non-overlapping tubules per animal were evaluated by two blinded observers; inter-observer agreement was assessed by Cohen’s κ/ICC. Only the cauda epididymis is presented in Figure 5 because this region exhibited the most pronounced structural alterations in response to the high-fat diet. As the primary site of sperm storage, the cauda is more suscepti ble to oxidative damage, mitochondrial dysfunction, and apoptosis, which aligns with the functional impairments observed in the HFD group. In contrast, histological changes in the caput were minimal and did not differ significantly between groups; therefore, caput images were not included to avoid redundancy (Figure 7).

### 3.8 Comparative analysis of LC3B, LC3B+Grp75, and p62 expression levels across groups

**FIGURE 8:**
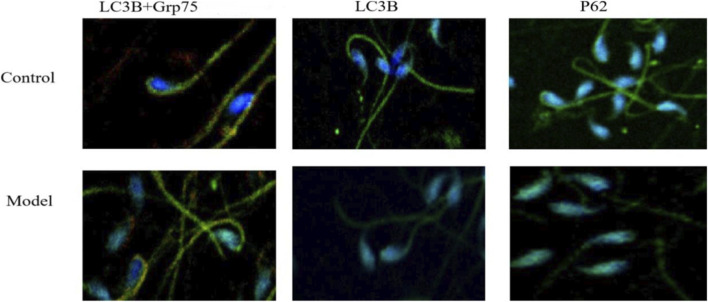
Immunofluorescence of LC3B, p62, and LC3B+Grp75 co-localization (×600). Sperm smears fixed in 4% PFA, permeabilized, and blocked with 5% BSA. Primary antibodies (LC3B, p62, Grp75; 1:200) were applied overnight at 4 °C, followed by FITC-conjugated secondary (1:500) and DAPI nuclear stain. Images captured on confocal microscope (Leica TCS SP8). Scale bar: 10 µm. Quantification of mean fluorescence intensity performed in ImageJ. *P < 0.05 versus CD.

LC3B is a classical marker of autophagosome formation, Grp75 is a mitochondrial chaperone protein involved in mitophagy, and p62 is an essential adaptor that links ubiquitinated substrates to the autophagic machinery. The combined analysis of these three markers facilitates the evaluation of general autophagy levels, mitophagy activity, and autophagic flux under high-fat diet conditionsImmunofluorescence assays were conducted to evaluate the expression levels of LC3B, LC3B+Grp75, and p62 proteins. The findings indicate that expression levels of LC3B and LC3B+Grp75 were significantly elevated in the HFD group compared to the CD group. Conversely, the expression of p62 was lower in the HFD group relative to the CD group. These differences were statistically significant (*P* < 0.05), underscoring the impact of dietary composition on autophagic markers. Refer to Table 4 and Figure 8 for a detailed presentation of these findings.

**TABLE 4:** Comparison of the expression levels of LC3B, LC3B+Grp75, and p62 in mice in each group ( $\bar{x}$ ±s).

Clusters	LC3B	LC3B+Grp75	p62
CD group (n = 3)	96.330 ± 31.500	15.670 ± 3.055	156.000 ± 32.600
HFD group (n = 3)	191.700 ± 28.150	98.670 ± 29.700	72.670 ± 11.850
*t*	3.909	4.814	4.161
*P*	0.0174	0.0086	0.0141

Data are expressed as mean ± standard deviation ( $\bar{x}$ ±s). Statistical analyses between groups were conducted using an independent-samples t-test. t represents the test statistic, and P denotes the corresponding probability value. *P* < 0.05 was considered statistically significant.

### 3.9 Outcomes of the TUNEL assay in mouse semen sections

**FIGURE 9:**
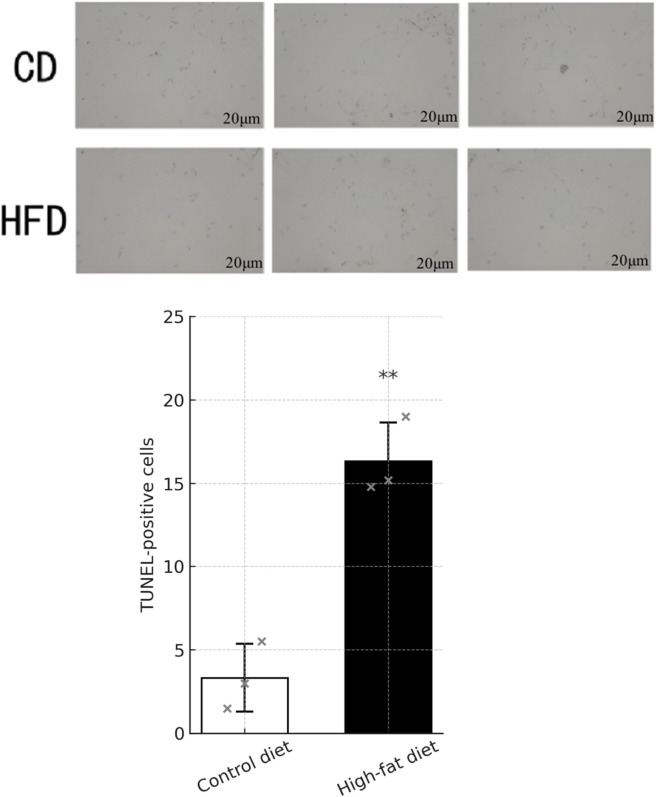
TUNEL assay of sperm apoptosis and quantitative analysis. Representative light-microscopic images (×100) of TUNEL staining in sperm smears from Control diet and High-fat diet mice. Apoptotic nuclei are visualized as brown-stained cells. Smears were fixed in 4% paraformaldehyde (30 min), permeabilized with 0.3% Triton X-100 (5 min), and endogenous peroxidase activity quenched with 0.3% H_2_O_2_/PBS (20 min). TdT-enabled incorporation of biotin-dUTP and subsequent Streptavidin-HRP/DAB development (5 min) were performed per the Roche *In Situ* Cell Death Detection Kit protocol, followed by hematoxylin counterstain. Quantification of TUNEL-positive cells. Five random fields per smear (n = 3 mice per group) were imaged under light microscopy (×100) and brown-stained nuclei counted. Data are presented as mean ± SD; error bars are drawn as “T”-shaped lines above each bar. Statistical significance was determined by unpaired two-tailed t-test (**P < 0.01 vs. Control diet).

Demonstrated a notable reduction in the presence of brown-stained cells within the semen sections of the high-fat diet (HFD) group compared to the control diet (CD) group. This observation is potentially linked to the decreased spermatozoa count observed in the HFD group. The distinct difference in apoptosis markers between the two dietary groups highlights the potential impact of diet on sperm viability. These results are visually represented in Figure 9 and Table 5.

**TABLE 5:** Quantitative analysis of TUNEL-positive cell count ( $\bar{x}$ ±s).

Groups	The number of TUNEL-positive cells
CD group	4.83 ± 1.17
HFD group	15.50 ± 2.43
*t*	6.536
*P*	0.0023

Data are presented as mean ± standard deviation ( $\bar{x}$ ±s). Statistical differences between the control and high-fat diet groups were analyzed using the independent-samples t-test. The t value represents the test statistic, and P denotes the corresponding probability value. A *P* < 0.05 was considered statistically significant.

### 3.10 Apoptosis assay findings in mice

**FIGURE 10:**
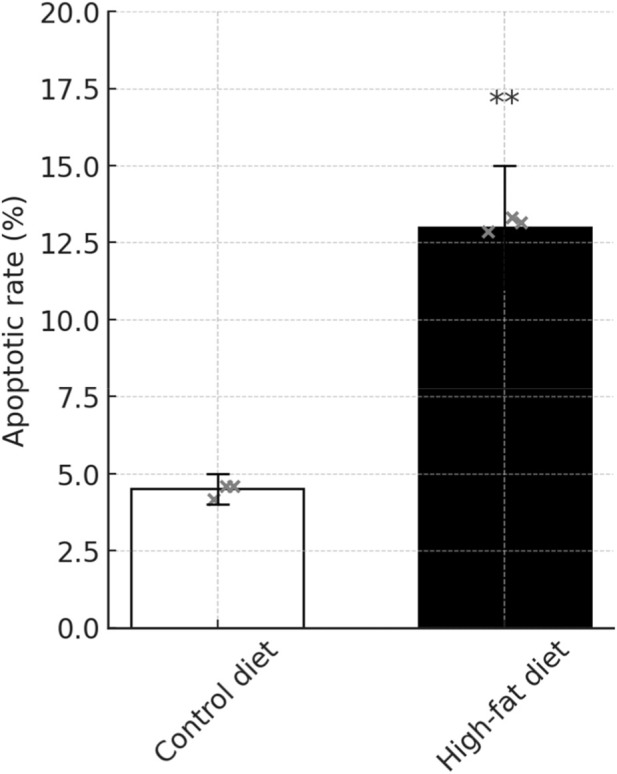
Flow cytometry analysis of sperm apoptosis. Spermatozoa were stained with Annexin V-FITC and propidium iodide (PI) following kit instructions (BD Pharmingen). Samples (n = 3 per group) were analyzed on a BD FACSCalibur; early and late apoptotic populations quantified using FlowJo. **P < 0.01 versus CD.

**FIGURE 11:**
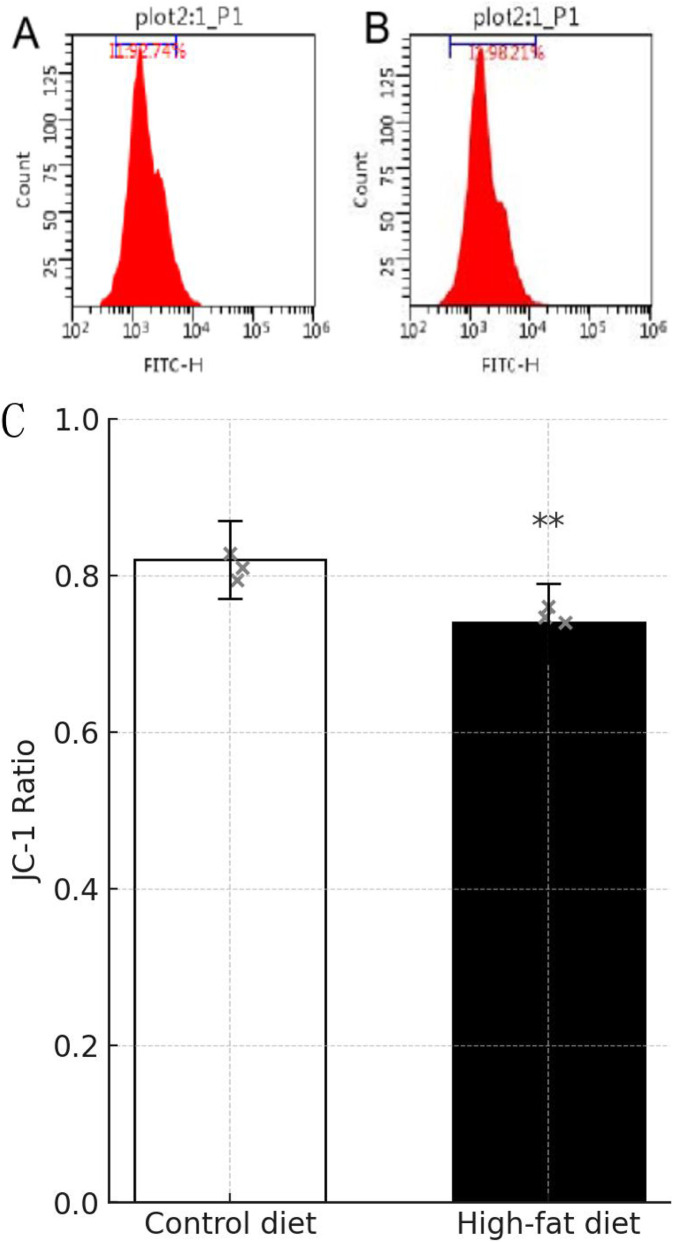
JC-1 analysis of mitochondrial membrane potential in mouse epididymal spermatozoa. **(A,B)** Following kit protocol (Beyotime), spermatozoa (n = 3 per group) were incubated with JC-1 dye and analyzed on a microplate reader and by fluorescence microscopy. Ratio of red (aggregate) to green (monomer) fluorescence was calculated. **(C)** Note: A: CDt group; B: HFD groupMitochondrial membrane potential was assessed in sperm collected from mice fed a control diet (CD) or high-fat diet (HFD) using the JC-1 dye assay. Briefly, sperm samples were incubated with 2 μM JC-1 (Beyotime, China) at 37 °C for 30 min in the dark. Following incubation, samples were washed and analyzed immediately using a fluorescence microplate reader (excitation/emission: 485/530 nm for monomers and 525/590 nm for aggregates). The JC-1 ratio (red/green fluorescence) reflects MMP levels. A decrease in the red/green fluorescence ratio indicates mitochondrial depolarization (n = 3 per group). **P < 0.01 vs. CD group.

**FIGURE 12:**
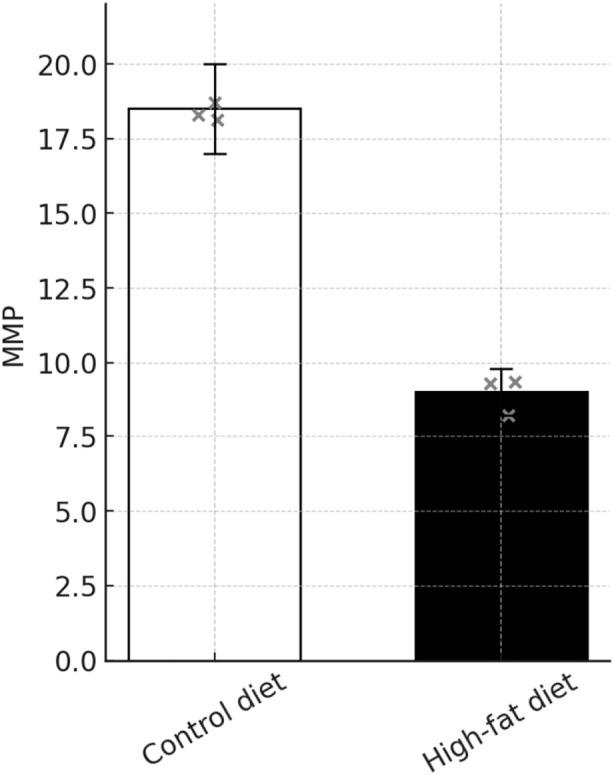
High-fat diet reduces mitochondrial membrane potential (MMP) in mouse testicular tissue. MMP changes were evaluated using a JC-1 mitochondrial membrane potential assay kit (Beyotime, China). Freshly isolated testicular tissues were enzymatically digested to prepare single-cell suspensions, adjusted to 1 × 10^6^ cells/mL, and incubated with JC-1 working solution at 37 °C for 20 min. After washing twice with PBS, the red/green fluorescence intensity ratio was measured using a flow cytometer (BD Accuri C6). The ratio of JC-1 aggregates (red fluorescence, high MMP) to monomers (green fluorescence, low MMP) reflects the MMP level. HFD significantly decreased MMP in testicular cells, indicating mitochondrial dysfunction (n = 3, **P < 0.01). Note: **P < 0.01 vs. control group.

**FIGURE 13:**
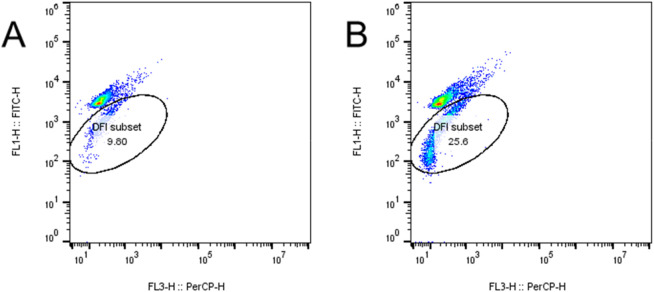
Sperm DNA fragmentation index (DFI) by flow cytometry. **(A)** Representative DFI scatter plot of the Control Diet (CD) group **(B)** Representative DFI scatter plot of the High-Fat Diet (HFD) group. The circled region indicates the DFI‐positive sperm population, and the percentage represents the proportion of sperm with fragmented DNA. Sperm DNA fragmentation w as measured using DFI kit (Cell Biolabs) by flow cytometry (BD FACSCalibur). DFI (%) represents the percentage of sperm with fragmented DNA (n = 3 per group). Data are mean ± SD; ***P < 0.001 versus CD.

These studies collectively indicate a significant effect of the high-fat diet on cellular functions, characterized by an elevation in apoptosis rates, increased mitochondrial membrane damage, and heightened levels of cellular ROS and DFI in spermatozoa. Detailed data supporting these findings are presented in Tables 6–9. Figures 10–13 JC-1 staining confirmed the reduction in mitochondrial membrane potential observed in the HFD group. Compared with the control group, the red/green fluorescence ratio was significantly decreased (*P* < 0.05), Quantification of mitochondrial membrane potential (MMP) in sperm using JC-1 stainin Figure 13. Although the differences in JC-1 fluorescence are not visually striking in the raw images, this is expected because JC-1 staining has limited visual sensitivity to mild changes in mitochondrial membrane potential. In the HFD group, a subtle reduction of red aggregates (high ΔΨm) and a slight increase in green monomers (low ΔΨm) can be observed, although the shift is modest. This visually subtle pattern is more accurately captured by the quantitative red/green ratio analysis.

**TABLE 6:** Standard deviation of semen apoptosis results by group ( $\bar{x}$ ± s).

Clusters	Mean ± standard deviation ( $\bar{x}$ ± s)
CDgroup (n = 3)	4.513 ± 0.217
HFD group (n = 3)	12.77 ± 2.452
*t*	5.807
*P*	0.0044

Data are presented as mean ± standard deviation ( $\bar{x}$ ±s). Statistical differences between the control and high-fat diet groups were analyzed using the independent-samples t-test. The t value represents the test statistic, and P denotes the corresponding probability value. A *P* < 0.05 was considered statistically significant.

**TABLE 7:** Standard deviation of changes in semen mitochondrial membrane potential by group ( $\bar{x}$ ± s).

Clusters	Mean ± standard deviation ( $\bar{x}$ ± s)
CD group (n = 3)	18.77 ± 1.823
HFD group (n = 3)	9.107 ± 0.752
*t*	8.485
*P*	0.0011

Data are expressed as mean ± standard deviation ( $\bar{x}$ ±s). Statistical analyses between the control and high-fat diet groups were performed using the independent-samples t-test. The t value represents the test statistic, and *P* denotes the corresponding probability value. A P < 0.05 was considered statistically significant.

**TABLE 8:** Standard deviation of semen ROS changes by group ( $\bar{x}$ ± SD).

Clusters	Mean ± standard deviation ( $\bar{x}$ ± s)
CD group (n = 3)	1.062 ± 0.083
HFD group (n = 3)	2.264 ± 0.014
*t*	24.68
*P*	<0.0001

Correlations were analyzed using Pearson’s correlation test for normally distributed data and Spearman’s rank correlation test for non-normally distributed data. r represents the correlation coefficient, and P indicates the probability value. A *P* < 0.05 was considered statistically significant.

**TABLE 9:** Standard deviation of semen DFI changes by group ( $\bar{x}$ ± s).

Clusters	Mean ± standard deviation ( $\bar{x}$ ± s)
CD group (n = 3)	9.641 ± 0.284
HFD group (n = 3)	25.73 ± 0.260
*t*	125.4
*P*	<0.0001

Data are presented as mean ± standard deviation (x̄ ± s). Statistical differences between the control and high-fat diet groups were analyzed using the independent-samples t-test. The t value represents the test statistic, and P denotes the corresponding probability value. A P < 0.05 was considered statistically significant.

### 3.11 Alterations in lipid profiles and testosterone levels in mice

The analysis of lipid profiles, including total cholesterol (TC), triglycerides (TG), low-density lipoprotein (LDL), and high-density lipoprotein (HDL), revealed a significant increase in these parameters within the high-fat diet (HFD) group when compared to the control diet (CD) group. This difference was statistically significant (*P* < 0.05). Conversely, testosterone (T) levels were found to be significantly lower in the HFD group as opposed to the CD group, with this reduction also being statistically significant (*P* < 0.001). These findings are detailed in Table 10.

**TABLE 10:** TC, TG, LDL, HLD, T expression levels (Mean ± SD, n = 3).

Clusters	CD group	HFD group	*t*	*P*
TG (mmol/mL)	0.304 ± 0.011	0.5393 ± 0.036	6.189	0.0035
TC (μmol/dL)	74.52 ± 2.482	147.4 ± 5.006	13.05	0.0002
LDL (mmol/L)	1.216 ± 0.048	1.610 ± 0.043	7.410	0.0018
HDL (mmol/L)	1.305 ± 0.026	1.592 ± 0.050	5.031	0.0073
T (ng/mL)	43.88 ± 0.513	25.19 ± 0.574	24.28	<0.0001

Correlations were analyzed using Pearson’s correlation test for normally distributed data and Spearman’s rank correlation test for non-normally distributed data. r represents the correlation coefficient, and P denotes the probability value. A P < 0.05 was considered statistically.

### 3.12 Testicular oxidative stress markers (ROS, MDA, SOD)

**FIGURE 14:**
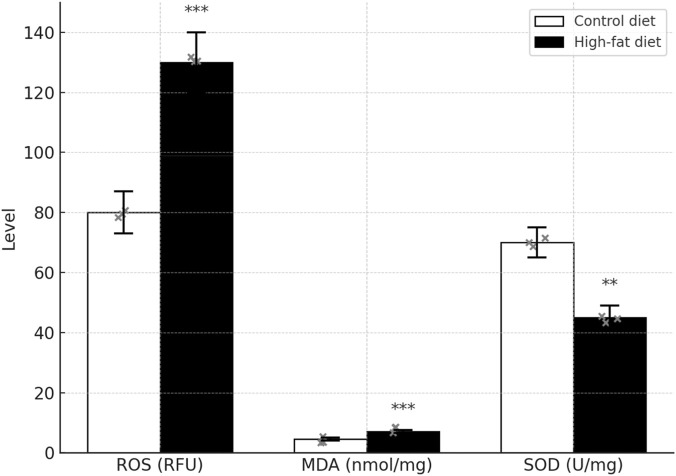
Testicular oxidative stress markers (ROS, MDA, SOD). Single-cell suspensions of testicular tissue were prepared by enzymatic digestion (1:10 w/v trypsin, 37 °C, 30 min), filtered through a 400-mesh nylon sieve, and washed twice with PBS (2,000 × g, 4 °C, 3 min). All assays were performed in triplicate (n = 3 per group) and normalized to protein concentration (BCA assay). *P < 0.05 versus Control diet; **P < 0.01 versus Control diet.

Compared with the control diet (CD) group, the high-fat diet (HFD) group showed significantly elevated ROS and MDA levels (*P* < 0.05) and a significantly reduced SOD level (*P* < 0.05) Figure 14.

### 3.13 Comparison of autophagy-related proteins

**FIGURE 15:**
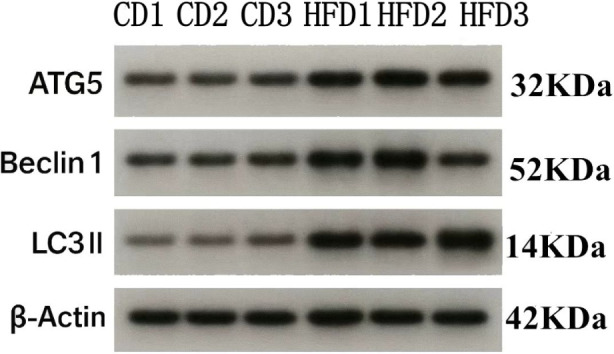
Autophagy-related protein expression in testicular tissue. Total protein was extracted from testicular samples of Control diet (n = 3) and High-fat diet (n = 3) mice in ice-cold RIPA buffer containing 1 mM PMSF, lysed on ice for 30 min, and centrifuged at 12,000 × g, 4 °C for 15 min. Protein (20 µg per lane) was separated on 12% SDS–PAGE (80 V stacking, 120 V resolving) and transferred to PVDF membranes (350 mA, 90 min). Membranes were blocked in 5% BSA-TBST (0.1% Tween-20) for 1 h at room temperature, then incubated overnight at 4 °C with primary antibodies against ATG5 (1:1,000, CST #12994), Beclin-1 (1:1,000, CST #3495), and LC3B (LC3II/LC3Ⅰ, 1:1,000, CST #3868). After three 5-min washes in TBST, membranes were incubated with HRP-conjugated secondary antibody (1:5,000) for 1 h at room temperature, washed again, and developed with ECL substrate for 2 min. Blots were imaged on a ChemiDoc MP system and quantified by ImageJ; target proteins were normalized to β-Actin (1:2,000, CST #3700).

**FIGURE 16:**
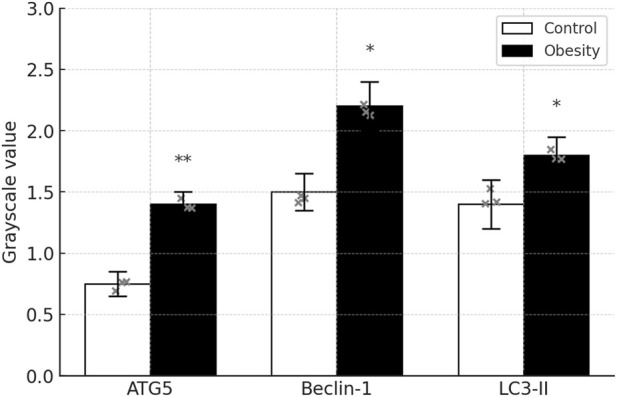
Quantification of autophagy markers Densitometric analysis of ATG5, Beclin-1 and LC3Ⅱ bands from Figure 15. Band intensities were measured in ImageJ, normalized to β-Actin, and expressed relative to Control diet. Data are mean ± SD (n = 3 per group); *P < 0.05, **P < 0.01 versus Control diet.

Compared with the control group, ATG5 expression was significantly increased in the high-fat diet group (*P* < 0.01); Beclin-1 and LC3-II levels were also significantly elevated in the high-fat diet group (*P* < 0.05) Figures 15, 16.

### 3.14 Comparison of key components of the apoptotic signaling pathway

**FIGURE 17:**
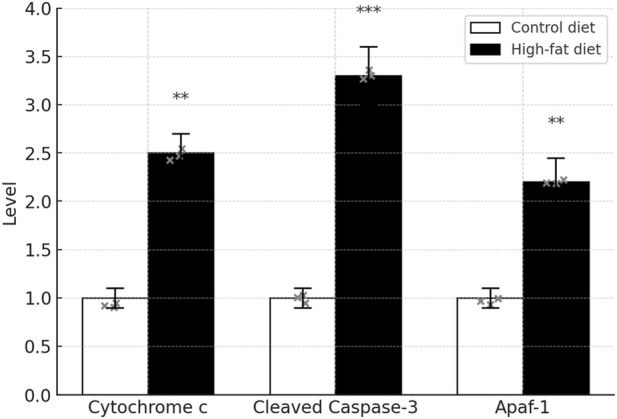
Key apoptosis signaling proteins in testicular cytosol. Cytosolic fractions were isolated from testicular homogenates using the Thermo Fisher cell fractionation kit (Cat. 89874). Protein concentrations were determined by BCA. Cytosolic proteins (20 µg per lane) were separated on 12% SDS–PAGE and transferred to PVDF membranes as above. Membranes were blocked (5% BSA–TBST, 1 h, RT) and incubated overnight at 4 °C with primary antibodies against Cytochrome c (1:1,000, CST #11940), Cleaved Caspase-3 (1:1,000, CST #9664), and Apaf-1 (1:1,000, CST #13072). After washing, membranes were incubated with HRP-secondary (1:5,000) for 1 h at RT, washed, and developed with ECL for 2 min. Blots were quantified in ImageJ and normalized to β-Actin. Data are mean ± SD (n = 3 per group); **P < 0.01 (versus Control diet).

Compared with the Control diet group, expression levels of cytosolic Cytochrome c, Cleaved Caspase-3, and Apaf-1 were markedly elevated in the High-fat diet group (*P* < 0.01) Figure 17.

### 3.15 Elevated 8-OHdG levels in sperm DNA of HFD-Fed mice

**FIGURE 18:**
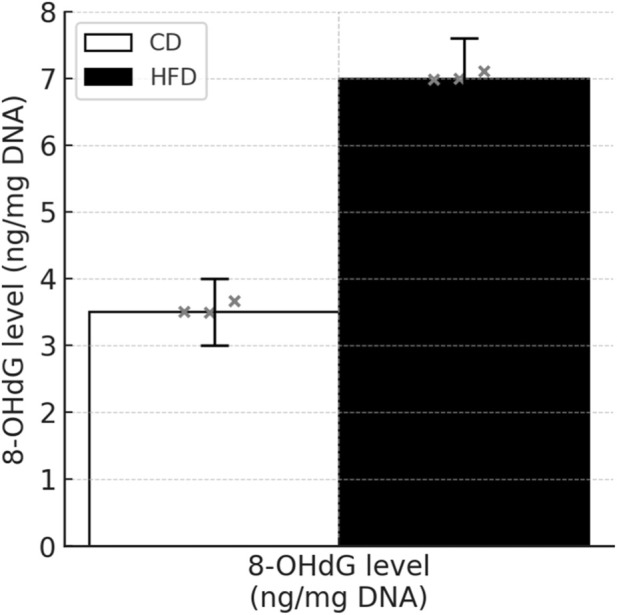
Levels of 8-hydroxy-2′-deoxyguanosine (8-OHdG) in sperm DNA of CD and HFD mice. Bar graphs represent the mean ± SD of 8-OHdG concentrations (ng/mg DNA) in spermatozoa from the control diet (CD) and high-fat diet (HFD) groups. Each dot indicates an individual mouse sample (n = 3 per group). HFD-fed mice exhibited significantly elevated 8-OHdG levels compared to controls (*P* < 0.001).

As shown in Figure 18, the concentration of 8-OHdG, a marker of oxidative DNA damage, was significantly higher in the HFD group compared to the CD group (CD: 3.21 ± 0.54 ng/mg DNA vs. HFD: 6.98 ± 0.73 ng/mg DNA, *P* < 0.001) Figure 18.

## 4 Discussion

As dietary trends evolve, lipid metabolism disorders are becoming more prevalent, disturbing the body’s functional equilibrium and promoting fat accumulation. Extensive research has shown that obesity negatively impacts male reproductive health (Han et al., 2023). Recent research indicates that obesity increases sperm DNA damage and may even affect the health and fertility of offspring through epigenetic mechanisms (Peel et al., 2023). Body Mass Index (BMI), a common obesity indicator, is widely reported to be inversely related to sperm quality in men (Klenov and Jungheim, 2014). Furthermore, evidence suggests that being overweight or obese is a risk factor for conditions such as oligozoospermia and azoospermia (Pini et al., 2020), with the obese male population showing a 3.5-fold increase in these conditions compared to normal-weight individuals (Jensen et al., 2008). Research from China also indicates that male mice on high-fat diets exhibit significantly reduced sperm concentration and viability, supporting findings in this study (Qi et al., 2023). Consequently, obesity might impair sperm quality by influencing apoptosis-related genes and lipid metabolism within the body.

C57BL/6J mice are commonly used in obesity research. This study assessed the effects of a high-fat diet (HFD) on sperm quality by feeding male mice with an HFD for 12 weeks. During the initial 1–3 weeks, no statistical difference in body weight was observed between HFD and control diet (CD) groups (*P* > 0.05). However, from weeks 3–6, the HFD group displayed a significantly greater weight increase than the CD group (*P* < 0.001). Moreover, from weeks 1–5, food intake in the CD group was higher than in the HFD group, suggesting that the HFD mice had reached obesity (*P* < 0.001). When evaluating female reproductive outcomes to infer male sperm quality, females paired with HFD-fed males did not become pregnant, whereas those with CD-fed males produced 17 offspring, indicating that obesity reduces male reproductive capability. Research on obesity and sperm quality is extensive but inconclusive; Ma et al. (2020) reported a negative correlation between BMI and sperm density/motility, and Ramaraju et al. (2018) found that high BMI reduces sperm volume, concentration, and motility while increasing malformations. Amoah et al. (2025) reported lower sperm density with higher BMI. Consistent with these studies, our results indicate lower total sperm count, forward motility, PR%, SC, and normal morphology percentage in the HFD group compared to the CD group. The NP% was higher in HFD males (*P* < 0.05), while IM% showed no difference (*P* > 0.05), indicating that obesity impairs sperm quality. Apoptosis plays a vital role in spermatogenesis, where about 75% of cells naturally undergo apoptosis, leaving 25% to develop (Zakariah et al., 2022). Our study found increased expression of pro-apoptotic genes (Bax, Caspase-3, -8, -9) and decreased anti-apoptotic genes (Bcl-2, Mn SOD) in the HFD group, suggesting an impairment in spermatogenesis. Apoptosis mechanisms are mediated by TNF-α receptors and caspase cascades, with Bcl-2 and Mn SOD involved in mitochondrial membrane permeability and cytochrome C release pathways (Ruiz-Valderrama et al., 2025; Wei et al., 2024). Additionally, autophagy-related gene expression (Bax, Beclin 1, Caspase-8, -9, LC3B/LC3A, LC3B/β-Actin) was elevated in the HFD group, suggesting suppressed autophagy and reduced sperm quality. Electron microscopy showed LC3B-II and autophagic vesicles in sperm cytoplasm, supporting autophagy’s role in sperm maturation. HE staining and immunofluorescence confirmed alterations in testicular tissue and spermatogenic cells in HFD mice. This study highlights the detrimental impact of a high-fat diet on male fertility, emphasizing the relationship between obesity, apoptosis, and autophagy in sperm quality and spermatogenesis. In line with these phenotypic changes, the region-specific transcriptional response observed along the male reproductive tract provides important mechanistic insight into how HFD disrupts sperm production and maturation. In the epididymal caput, upregulation of Beclin-1 and LC3B together with downregulation of p62 suggests an activated autophagic flux, which may initially represent an adaptive attempt of epithelial and supporting cells to clear damaged organelles and misfolded proteins under metabolic stress. However, persistent HFD exposure likely drives autophagy beyond a protective threshold, leading to excessive turnover of cellular components and disturbance of the finely tuned luminal microenvironment required for early sperm maturation. By contrast, in the cauda epididymis, the predominance of pro-apoptotic signals (increased Caspase-3, Cytochrome c and Bax with reduced Bcl-2) indicates that terminal storage sites for sperm are more prone to apoptotic elimination of germ cells and mature spermatozoa, which may directly decrease the pool of functionally competent sperm and aggravate DNA fragmentation. In the testis, concurrent elevation of Beclin-1, ATG5, Caspase-3 and Apaf-1 points to a crosstalk between autophagy and mitochondrial apoptosis in response to HFD-induced oxidative stress. Autophagy is generally thought to remove damaged mitochondria and limit ROS generation, but when ROS overload is sustained and mitophagy is insufficient or dysregulated, mitochondrial outer membrane permeabilization and cytochrome c release are facilitated, ultimately activating the Caspase cascade and germ-cell loss. Taken together, these region-specific molecular signatures support a model in which HFD first induces an “adaptive–maladaptive” shift of autophagy in the epididymis and testis, and then progressively tilts the balance toward apoptosis, culminating in impaired spermatogenesis, defective epididymal maturation and reduced fertility.

The mitochondrial structure, comprising both inner and outer membranes, is crucial for ATP synthesis and for sustaining a highly negative mitochondrial membrane potential, essential to mitochondrial function. This membrane potential serves as a core indicator of mitochondrial health and plays a significant role in evaluating sperm quality, as studies show a link between reduced mitochondrial membrane potential and lower sperm quality (Marcheri et al., 2002). Our findings suggest that a high-fat diet impairs cellular function by promoting apoptosis, damaging mitochondrial membranes, and increasing reactive oxygen species (ROS) and DNA fragmentation index (DFI) levels in sperm, indicating mitochondrial dysfunction in mice fed a high-fat diet. In a study involving 483 infertile couples, Baydilli et al. (2020) discovered in infertile couples, higher BMI in men is associated with lower testosterone levels and elevated estradiol levels, along with decreased semen quality, indicating that obesity affects male reproductive function through endocrine mechanisms. In line with these observations, our study found increased total cholesterol (TC), triglycerides (TG), low-density lipoprotein (LDL), and high-density lipoprotein (HDL) in the high-fat diet (HFD) group compared to the control diet (CD) group, alongside a marked decline in T levels (*P* < 0.001). These findings suggest that lipid imbalances may impair sperm quality due to fat accumulation in HFD-fed mice, affecting hormone regulation from the hypothalamic-pituitary-testicular axis and modifying the spermatogenic environment. This imbalance decreases spermatogonial cell count, reduces testosterone production by interstitial cells, and converts testosterone into estradiol within fat cells, thereby compromising sperm quality. Additionally, cholesterol is essential for maintaining cell membrane integrity, and alterations in membrane lipids can compromise cellular function. Simón et al. (2018) demonstrated that high-cholesterol diets in New Zealand male rabbits led to increased cholesterol in sperm cell membranes and decreased tyrosine-phosphorylated proteins, negatively impacting membrane permeability, fluidity, and substance exchange, ultimately reducing sperm quality. In our study, the elevated lipid levels in the HFD group may impair cell secretion, interfere with spermatogenic and supporting cell communication, and disrupt spermatogenesis and maturation.

Analysis of testicular tissues using transmission electron microscopy in this study identified a reduction in mitochondrial numbers and marked mitochondrial vacuolation in interstitial cells in the HFD group, along with reduced electron density in nuclear chromatin and vacuole formation. These findings suggest that high-fat diets result in mitochondrial dysfunction within testicular cells, leading to decreased reproductive capability in mice, thus highlighting the harmful cellular and molecular effects of dietary habits on male fertility. Moreover, studies have also shown that exogenous chemicals can induce similar mitochondrial and nuclear alterations via estrogen receptor–dependent signaling and oxidative stress (Han et al., 2023; Peel et al., 2023).

In contrast to recent studies that have primarily focused on testicular apoptosis or conventional oxidative damage markers (Liu et al., 2024; Allam et al., 2022; Sun et al., 2023), our study offers several novel insights. First, we are the first to jointly assess reactive oxygen species (ROS), mitochondrial membrane potential, and sperm DNA fragmentation index (DFI), thereby establishing a comprehensive injury axis. Second, we report for the first time the co-localization of LC3B and Grp75 in sperm cells, suggesting potential mitophagy dysfunction, which is further supported by downregulation of p62 expression. Finally, by correlating ultrastructural alterations observed via transmission electron microscopy with key molecular markers such as Bax and LC3B, we bridge the gap between morphological changes and underlying functional mechanisms—an approach not previously addressed. In addition to elevated ROS levels and increased DNA fragmentation, we further observed a significant rise in 8-OHdG levels in the spermatozoa of HFD-fed mice. As a well-recognized biomarker of oxidative DNA damage, 8-OHdG reflects direct injury to nuclear DNA and is closely associated with male infertility. Previous studies have shown that increased 8-OHdG levels are linked to reduced sperm motility and fertilization capacity (Lee et al., 2022). Therefore, our findings suggest that HFD-induced oxidative stress not only impairs mitochondrial and apoptotic pathways but also causes direct genetic damage, further compromising sperm function.

In summary, this study suggests that a high-fat diet negatively affects sperm quality, likely via a network of genetic and biochemical interactions. Findings show increased expression of genes related to apoptosis and autophagy, including Bax, Beclin 1, Caspase-3, Caspase-8, Caspase-9, LC3B/LC3A, and LC3B/β-Actin, as well as elevated levels of total cholesterol (TC), triglycerides (TG), LDL, and HDL. At the same time, lower expression of anti-apoptotic and antioxidant genes such as Bcl-2, Mn SOD, and p62, along with reduced testosterone (T) levels, further highlights the comprehensive effects of a high-fat diet on reproductive health. Increased apoptosis, mitochondrial damage, higher reactive oxygen species (ROS), and an elevated sperm DNA fragmentation index (DFI) collectively contribute to impaired reproductive function in male mice. This analysis underscores the importance of further research into dietary effects on molecular mechanisms affecting male fertility, suggesting potential therapeutic and lifestyle approaches to counteract obesity-related reproductive health issues.

## Data Availability

The original contributions presented in the study are included in the article/supplementary material, further inquiries can be directed to the corresponding author.
